# Effectiveness of an Integrated Care Package for Refugee Mothers and Children: Protocol for a Cluster Randomized Controlled Trial

**DOI:** 10.2196/25047

**Published:** 2021-05-04

**Authors:** Zunayed Al Azdi, Khaleda Islam, Muhammad Amir Khan, Nida Khan, Amna Ejaz, Muhammad Ahmar Khan, Azza Warraitch, Ishrat Jahan, Rumana Huque

**Affiliations:** 1 ARK Foundation Dhaka Bangladesh; 2 Association for Social Development Lahore Pakistan

**Keywords:** mental health, refugee health, early childhood development, Rohingya, Bangladesh, community health care, community health worker

## Abstract

**Background:**

Thousands of Rohingya refugee mothers at the world’s largest refugee camp located in Bangladesh are at risk of poor mental health. Accordingly, their children are also vulnerable to delayed cognitive and physical development.

**Objective:**

The aim of this study is to evaluate the effectiveness of an integrated care package in reducing the prevalence of developmental delays among children aged 1 year and improving their mothers’ mental health status.

**Methods:**

This is a parallel, two-arm, single-blind, cluster randomized controlled trial (cRCT). A total of 704 mother-child dyads residing at the Kutupalong refugee camp in Cox’s Bazar, Bangladesh, will be recruited from 22 clusters with 32 mother-child dyads per cluster. In the intervention arm, an integrated early childhood development and maternal mental health package will be delivered every quarter to mothers of newborns by trained community health workers until the child is 1 year old. Our primary outcome is a reduction in the prevalence of two or more childhood developmental delays of infants aged 1 year compared to the usual treatment. The secondary outcomes include reduced stunting among children and the prevalence of maternal depression. We will also assess the cost-effectiveness of the integrated intervention, and will further explore the intervention’s acceptability and feasibility.

**Results:**

At the time of submission, the study was at the stage of endpoint assessment. The data analysis started in December 2020, and the results are expected to be published after the first quarter of 2021.

**Conclusions:**

This study will address the burden of childhood developmental delays and poor maternal mental health in a low-resource setting. If proven effective, the delivery of the intervention through community health workers will ensure the proposed intervention’s sustainability.

**Trial Registration:**

ISRCTN Registry ISRCTN10892553; https://www.isrctn.com/ISRCTN10892553

**International Registered Report Identifier (IRRID):**

DERR1-10.2196/25047

## Introduction

Rohingya refugees settled in Bangladesh are one of the largest groups of refugees in the world [1]. Cumulatively, by 2019, approximately 911,566 Rohingya refugees settled in both refugee camps designated by the government as Forcibly Displaced Myanmar Nationals [2]. Among the camp residents, 52% are women, 23% of whom are within reproductive age [3].

These refugees residing in the camps live in drastic situations and suffer from hunger, poverty, lack of safety, and appropriate access to health services [3]. The mental health status of Rohingya refugee women has been reported to be poor, which could be due to prolonged exposure to violence, trauma, and stress of living under terrible circumstances [4]. Maternal mental health is a crucial factor in ensuring healthy child development [5]. However, women who have experienced a traumatic event in their lives, as is the case for refugees, are at higher risk of postpartum depression, which can impact the growth and nutrition [6] and development of their child [7], as childhood development is mediated by mothers’ responsive feeding and caregiving skills. Moreover, prolonged exposure to psychosocial risks such as maternal depression, violence, and lack of stimulation can profoundly affect children’s health and cognitive development under the age of 2 years [8]. Studies have shown that at least 2% of the total refugees who are children (approximately 17,200) aged less than 1 year [9] are at risk of delayed development at the refugee camps.

Health care barriers faced by refugees continue to increase the risk of delayed child development and poor mental health for women [10]. Only 10 hospitals currently serve refugee settlements with an allocation ratio of 1 per 130,000 people. The health care systems are overburdened, short-staffed, and lack the necessary resources and infrastructure to provide adequate care, and have been reported to show significant gaps in treatment available for mental health and child development care. These health care challenges indicate the need to evaluate a service delivery model for early child development that will help support the health care providers with effective, scalable, and cost-effective alternatives to promote the child development and maternal mental health of refugees [11]. Integrated childhood development care within maternal, neonatal, and child health services has already proven to be effective in preventing developmental delays for children 2 years of age in a similar context [12,13].

To address the challenges highlighted above, this study has the objectives to: (1) evaluate the effectiveness of an integrated care package in reducing the prevalence of two or more developmental delays among infants aged 1 year and improving childhood stunting compared to the usual treatment, (2) evaluate the effectiveness of the integrated care package in reducing maternal depression, (3) explore the cost-effectiveness of the integrated care package in reducing childhood developmental delays, and (4) perform a mixed method process evaluation study to explore the acceptability and feasibility of the intervention for both the providers and participants.

## Methods

### Study Design, Settings, and Participants

We will use a parallel arm, single-blind, cluster randomized controlled trial (cRCT) design [14] to evaluate the integrated and contextualized package's effectiveness in reducing childhood developmental delays compared to the usual treatment.

The study will be performed in the Kutapalong Rohingya refugee camp located in Cox's Bazar, Bangladesh, selected based on its size and distance from the district city and ease of communication. The Kutupalong refugee settlement is a cluster of 20 camps, most of which are adjacent to each other. Each camp has definite boundaries and segments called “blocks.” Two blocks are combined to form a cluster for randomization in this study.

The research participants will be 704 mother-child dyads recruited from the 22 clusters in the Kutupalong refugee camp. The inclusion criteria for mother-child pairs are that the child should be less than or equal to 6 weeks old, live with their biological mother, had a gestational period of at least 36 weeks, and weighed at least 2.5 kilograms at birth. Children with congenital abnormalities and mothers that have to move out of the area during the study period will be excluded from the trial. Participation of mother-child dyads in the study will be required for 12 months.

### Procedures

#### Randomization and Masking

To minimize the risk of contamination between research participants, the randomization unit will be a cluster comprising two blocks. Blocks are geographical areas with defined boundaries in refugee camps. A sampling frame of eligible blocks within camps in the study site will be drawn before randomization using the population data and live birth record rates. Eligible clusters will be randomized before the recruitment of research participants from each cluster. The clusters taking part in the study will be randomized to the intervention or control arm by an independent statistician on a 1:1 allocation ratio. SAS PROC PLAN will be used to generate the randomization sequence code.

Given the nature of the intervention, it will be impossible to blind participants to the treatment allocation status. However, the assessment team, principal investigators, and the trial statistician will be blind to clusters’ allocation status.

#### Sample Size Calculations

For a two-sided hypothesis test with 22 clusters randomized at a 1:1 allocation ratio, and assuming an effect size of 0.35 with outcome proportions ranging from 34% to 20% for child development and from 30% to 15% for maternal depression, with 80% power, .05 significance, an intracluster correlation coefficient of 0.12, and accounting for 10% attrition, we will need 704 mother-child dyads (ie, 352 in each group), with 32 participants from each cluster on average. Findings from evidence synthesis indicate that early child development interventions usually yield small effect sizes [15], ranging from 0.2 to 0.4, per the Cohen criteria for effect sizes [16].

#### Package of Care in Intervention and Control Arms

The care package will be delivered by the community health workers (CHWs) identified from the selected clusters. CHWs having at least 10 years of formal education and willing to contribute to the community will be preferred for collecting data and delivering the intervention.

For standardization of research results, the control arm will be strengthened by providing a 2-day training to CHWs on recruitment of the mother-child dyads, administration of outcome measures, record-keeping, log maintenance, compliance, and communication. They will also be trained on taking anthropometric measurements to record the children’s height, weight, and mid-upper arm circumference (MUAC) every quarter. These inputs will be the same for the control and intervention arms.

In addition to the procedures mentioned above, CHWs in the intervention arm will be provided an additional 2 days of training on delivering the intervention to mothers. They will be trained on using necessary counseling skills while interacting with the mothers, such as empathy, rapport-building, trust, sympathy, privacy, mindfulness, and suggestion. They will also learn how to deliver counseling sessions to participants using a pictorial training flipbook with educative messages.

**Figure 1:**
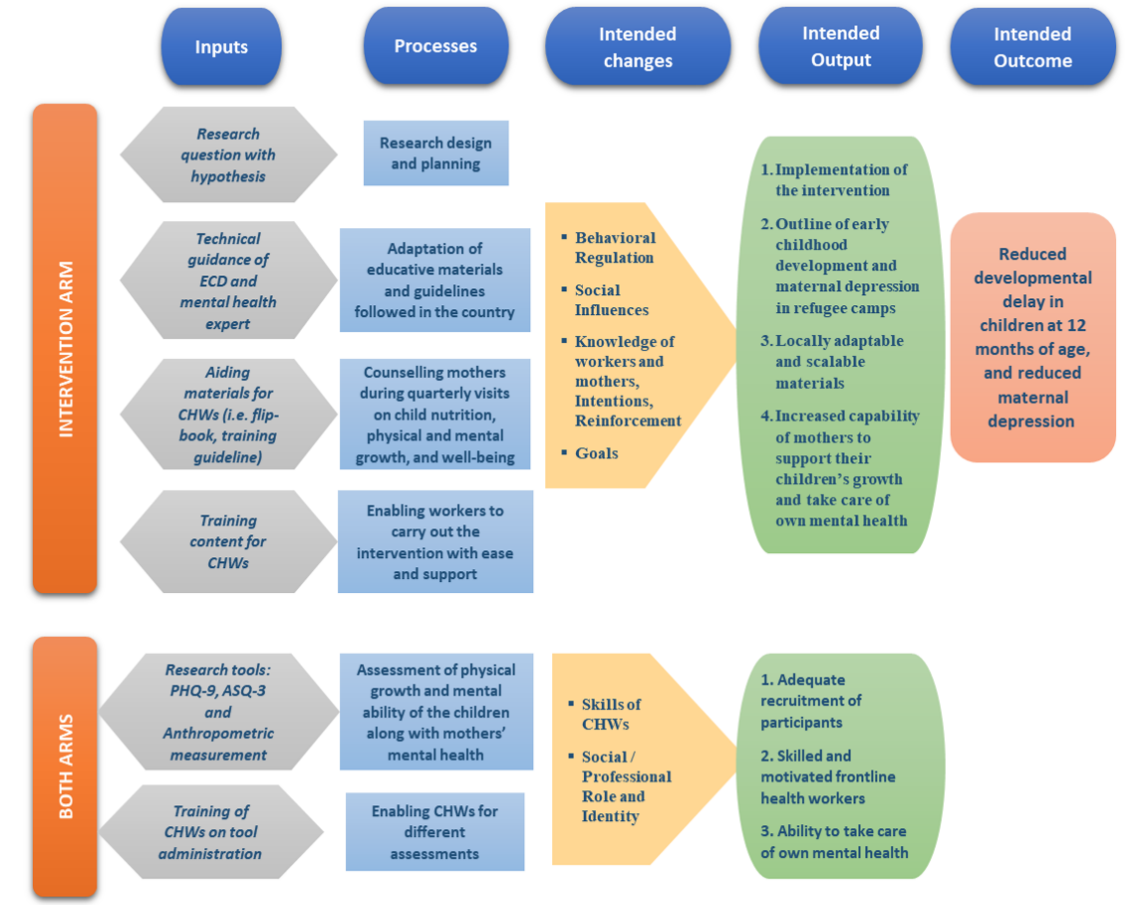
Logic model for intervention mechanism. CHW: community health worker; ECD: early childhood development; ASQ-3: Ages and Stages Questionnaire third edition; PHQ-9: Patient Health Questionnaire-9.

The integrated care package delivered in the intervention arm has been adapted and contextualized in consultation with international early childhood development and mental health experts. A logic model describing the intervention mechanism is presented in Figure 1.

A total of four counseling sessions will be delivered to mother-child dyads by CHWs in the intervention arm to promote early child development and maternal mental health. The counseling sessions will focus on the child’s cognitive and physical development, and the mother’s mental health based on a few key messages (see Table 1). The counseling contents are developed in consultation with technical experts, supported by a pictorial flipbook that has been modified according to the local context and translated into Burmese to be consistent in delivering the messages. The flipbook pictures are self-explanatory, and the CHWs will explain each of the pictures regardless of the mother’s ability to read the text of the flipbook. Each session will take at least 10-15 minutes.

**Table 1:** Key counseling messages and their delivery time for the intervention arm according to child age.

Theme		Key messages to the mother
**First quarter: 0-6 weeks old**		
	Nutrition	Frequent and exclusive breastfeeding, avoiding intake of other food items, and timely immunization are essential to the child’s health
	Mental ability	Ensure your presence and attention toward the child by caressing, talking, and looking at them with affection and a smile
	Physical ability	To improve the child’s physical ability, encourage the movement of their body parts
	Mother’s health	Eating full meals thrice a day, using iodized salt, and taking rest are essential to the mother and child’s health
**Second quarter: 3 months old**		
	Mental ability	Play with the child and make them aware of different parts of the face, sounds, and colors
	Physical ability	Increase the movement of different body parts of the child for the development of their physical health
**Third quarter: 6 months old**		
	Nutrition	Roti, rice, curry, and other food items at home (eg, kheer, mashed fruits) are important components of a child’s soft food
	Diet	After cooking properly with necessary ingredients, smash them and prepare soft food for your child
	Protecting health	Clean/wash utensils, regularly wash your hands, and cover food to prevent the child from becoming ill
	Mental ability	Encourage the child to pronounce words, identify facial parts, be with other children of the same age group, and find hidden items
	Physical ability	Encourage the child to use different body parts for improved physical ability
**Fourth quarter: 9 months old**		
	Mental ability	Encourage the child to participate in daily activities/identify items/follow instructions/find hidden items
	Physical ability	Encourage the child to use different body parts for improved physical ability
	Maternal mental health	Make a routine to pray, share your emotions with a trustworthy person, and make time for yourself and your mental well-being

#### Data Collection and Outcomes

Our primary outcome is the reduction in the prevalence of two or more childhood developmental delays of infants aged 1 year compared to the usual treatment, which will be measured by the Ages and Stages Questionnaire (ASQ) 3rd edition [17]. The ASQ is a brief, valid, and reliable measure of childhood development that is widely used to assess childhood developmental difficulties [18]. The ASQ has also been widely used in lower-middle-income countries and has been reported to be culturally valid [19,20]. It has 30 items and consists of 5 subscales to measure communication skills, fine motor, gross motor, problem-solving, and personal-social skills.

Secondary outcomes include stunting and maternal depression. Children’s anthropometric data on height, weight, and MUAC will be collected as part of the delivery process by the CHWs every quarter.

Patient Health Questionnaire-9 (PHQ-9) will be used to measure maternal depression at the endpoint of the study by the trained external assessors. The PHQ-9 has 9 items, which are rated on a 3-point Likert scale of 0 (not at all) to 3 (nearly every day) [21,22].

### Project Evaluation

**Figure 2:**
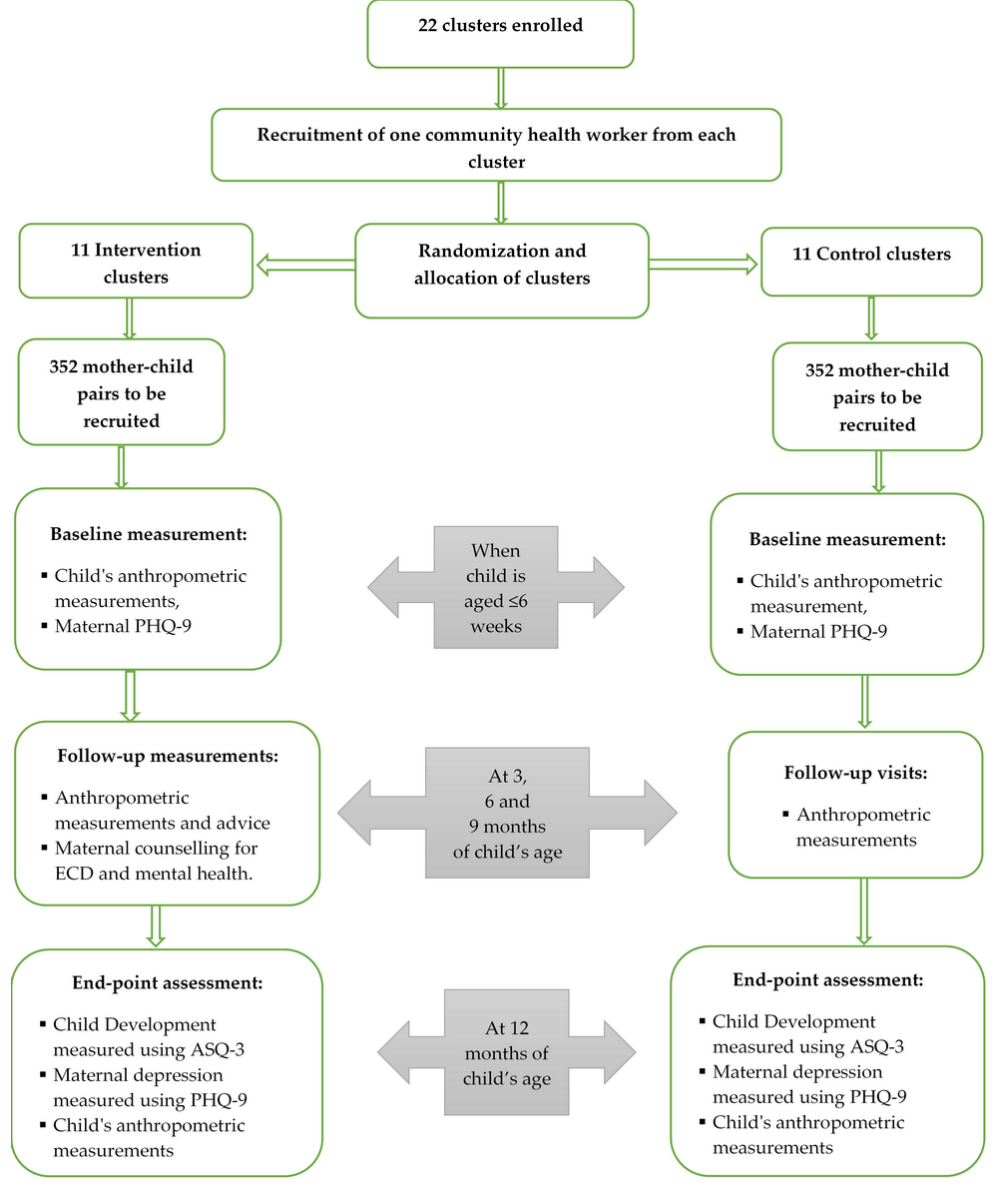
Trial flow. ECD: early childhood development; ASQ-3: Ages and Stages Questionnaire third edition; PHQ-9: Patient Health Questionnaire 9.

The trial flow is given in Figure 2. The project implementation will be evaluated to understand the scalability and sustainability of the intervention. We will use the following evaluation methods: (1) process evaluation and (2) economic evaluation.

In process evaluation, the trial will be followed by a mixed methods approach following the Medical Research Council guideline [23]. Quantitative data on the trial’s implementation will be extracted from the study records. Simultaneously, the qualitative data will be collected via in-depth interviews with participants and providers to explore the intervention’s acceptability and feasibility.

An exploratory economic evaluation will be performed to assess the integrated early childhood development and maternal mental health program’s cost-effectiveness in refugee camps. Project budgets and expenditure reports will be used to estimate the costs of the intervention, followed by calculating the incremental cost-effectiveness ratio using World Health Organization guidelines [24].

### Statistical Analysis

The findings of the study will be reported following CONSORT guidelines for cRCTs [14]. The data will be entered regularly after receiving the paper forms from the field, and then checked for missing data before the next visit scheduled in the clusters, allowing researchers to communicate with the CHWs to address such issues. Furthermore, we will follow the recommended methods for treating missing data per the guidelines of the outcome measures being used. Finally, we will analyze data using intention-to-treat analysis that handles any missing data at the endpoint. Descriptive statistics will be calculated for outcome variables and baseline characteristics of participants according to treatment arm to ensure the comparability of all outcomes across arms. Adjusted analysis and subgroup analysis will be based on covariates determined at baseline.

Data will be analyzed using cluster trials with relatively few clusters in each arm in IBM SPSS Statistics version 23. Crude analysis to estimate cluster-level proportions will be used for categorical outcomes. An independent sample *t* test to calculate the absolute difference in outcome proportions between the two study arms at the endpoint will be calculated with 95% CIs and significance values. For continuous outcomes, cluster-level outcome values based on the mean outcome scores in each cluster will be calculated, and an independent *t* test will be used to estimate the treatment effect as the mean difference in the cluster level outcome values between the two arms (control and intervention) at the endpoint, with associated 95% CIs and *P* values. A two-stage method will be used to adjust for confounding variables using a logistic regression model for individual-level outcome data. We will then calculate the covariate-adjusted difference residuals for each cluster by calculating the mean difference between observed and predicted outcomes. Independent *t* tests will be used to estimate the covariate-adjusted treatment effect as the risk difference in the cluster-level difference residuals between the two arms, with associated 95% CI and *P* values. No interim analysis of outcomes is planned.

### Ethical Approval

Ethical approval for the study has been obtained from two government bodies in the country: (1) Bangladesh Medical Research Council for research under reference number BMRC/NREC/2016-2019/843 and (2) Refugee, Rehabilitation and Repatriation Commission for project implementation under reference number ShoTraProKa/RHU/ARK Foundation/13/2019/589.

## Results

During the submission of this paper, the study was at the stage of endpoint assessment. The analysis of data obtained from the field started in December 2020, and we expect to publish the study results after the first quarter of 2021.

## Discussion

The aim of this study is to address the health and economic burden of childhood developmental delays and maternal mental health by delivering a community-based integrated care package in Bangladesh’s refugee camps. Intervention delivery by the community health care volunteers will ensure the proposed intervention’s sustainability if proven useful in the context. To the authors’ knowledge, this study is the first to test an integrated care package for early childhood development and maternal mental health in refugee camps.

The intervention and its components were designed in consultation with international experts, collaborators, and primary health care specialists in Bangladesh. However, some anticipated challenges in implementing the intervention can be anticipated. First, retention of the project’s CHWs might be a challenge, as they continuously look for better income opportunities. In that case, repeated search of CHWs may be needed for clusters, and additional refresher training sessions may need to be organized. Second, the language barrier between the field coordinators and the CHWs may result in communication gaps, affecting intervention delivery; a translation expert might be used to address this challenge. Third, mothers of the intervention arm will be more familiar with the child’s development activities, creating recall bias during the endpoint assessment of a child’s development at 12 months. This issue may be addressed by performing on-site observations. The qualitative aspect of the process will help us to better understand the participants’ and providers’ challenges during implementation.

The study results will be used to achieve impact by being embedded within the country’s health care system. Stakeholders at different levels will be engaged for the maximum impact of maternal mental health on childhood development. Moreover, general practitioners in emergency settings such as those working inside the refugee camps, set up by different national and international organizations, can adapt and integrate the approach with their “First 1000 Days” health interventions for better health outcomes of both mothers and children. Upon success, a similar intervention can be replicated in the host community with the help of the existing health workforce.

## Appendix

Multimedia Appendix 1 Peer-review report by the Canadian Institute of Health Research.

## Abbreviations

ASQ: Ages and Stages Questionnaire
CHW: community health worker
cRCT: cluster randomized control trial
MUAC: mid-upper arm circumference
PHQ-9: Patient Health Questionnaire 9
