# Supplementary material for: Effectiveness of an Integrated Care Package for Refugee Mothers and Children: Protocol for a Cluster Randomized Controlled Trial
Source: JMIR Res Protoc. 2021 May 4;10(5):e25047. doi: 10.2196/25047 (PMC8132976; doi:10.2196/25047)
Supplement: Multimedia Appendix 1 [file resprot_v10i5e25047_app1.pdf]

Canadian Institute of Health Research

Effectiveness of an integrated care package for Refugee mothers and children: a cluster randomized controlled trial

PEER REVIEW COMMENTS

Reviewer 1

This proposal put forward by a Bangladesh based NGO (ARK) and a Pakistan based NGO (ASD, which is also a previous/current recipient of a GCC awards) aims to adapt and conceptualize an existing ECD and maternal mental health care and support model (from Pakistan) for Rohingya refugee women and children dyads who are based in selected camps in Bangladesh. A key element is the training of local women (who are also refugees and in the camp) with at least a grade 10 level of education to serve as the providers of the ECD/parenting activities. This model has been developed and tested in Pakistan by ASD (and received funding from GCC). The main hypothesis is that the delivery of ECD and maternal mental health by modestly educated community providers is effective and feasible in a refugee camp context. Neither of the two organizations have any history/experience of working within refugee camps to date but see a clear need for contextualized approaches for ECD and maternal mental health - indeed they state there is a lack of any contextualized, evidence based models for refugee camp settings.

According to the proposal, some 750,000 refugees (13000 of whom are infants below 1yr) are in camps in the country and the numbers are growing and may exceed 1mio. The government has asked NGOs to provide assistance to the refugees.

They plan to review and adapt (contextualize) the existing content to understand provider and consumer perspectives to child development and maternal mental health. They they plan to develop/adapt a 2-day training for the proposed 12 community based care providers (who are meant to be drawn from the same refugee camps) who will implement the program with the target mothers (696 mother/child dyads). Each community-based provider will work with 60 mother/child dyads. A field supervisor/coordinator will conduct monthly performance meetings to understand progress, discuss issues/problems with the aim of improving the implementation. The monitoring and research plan is fairly clear with three key aspects: a cluster randomized control trial (22 clusters with 696 mother/child dyads), process evaluation of the contextualized model for ECD and a costing exercise to understand what costs might look like with scaling in future, if successful.

The teams, while not having worked in refugee camp settings before appear to have the skills and competencies in the key areas (ECD, maternal mental health, program implementation,

research). The experience of the GCC awardee from Pakistan is positive in that the approach funded by GCC is now to be adapted/contextualized to the camp for Rohingyas.

The potential of having an approach for ECD and for maternal mental health for the context of refugee camps in Bangladesh and elsewhere could be very positive and significant in terms of numbers. Their plans for interactions and reviews with a range of government, NGO and international funders should assist with dissemination of the approach and its effectiveness and therefore useful as part of a process for replication/scaling.

Key issues /Questions
The review of stakeholder perspectives at the start will be critical in the adaptation and contextualization process. This reader hopes that the planned process evaluation process continues to provide ongoing feedback in helping to refine and continue to shape the overall approach. It would be useful to understand a bit more of the organizations' thinking on how they might refine the content and process over the 2-year period.

The implementing partners plan to identify and train other women in the refugee camps with a grade 10 education to be the community providers of the ECD services. A potential concern here is that as refugees themselves, and therefore facing their own displacement, psychosocial distress, etc have the agencies considered what might be needed for these women in terms of support? It should be noted it is a positive feature to draw these community providers from the same communities with similar language, cultural, etc experiences. A second area of concern to this reader is the proposed 2-day training period for these community providers - during which content, presumably strategies/activities, planning and also use of some key monitoring tools needs to be relayed and understood. This reader hopes that follow up and refresher training might be provided but it is not very clearly stated except to mention monthly performance reviews.

It would seem important to consider what supports may be needed to ensure adequate understanding of the content, ability to deliver the same to mothers/children and ability to adequately carry out the monitoring with the selected tools. In addition, while the approach being adapted is in use in Pakistan, it was not entirely clear how successful or what impacts were being seen to date.

A further concern, which the implementing organizations may not be able to control but could negatively impact their implementation and research efforts, relates to the proposed 696 mothers/children who will be tracked over the two years and how stable (or not) the refugee population will be in the camps. Nonetheless, the importance of having culturally sensitive/contextualized approaches for use in refugee camps/emergency areas and the current situation of the Rohingya is clearly critical and requires feasible and cost effective ideas.

1. Impact

Feedback: the potential impact of this program beyond the specific target areas could be significant given the rising numbers of refugees from Myanmar as well as others in many other countries experience conflict, emergencies. This looks to be a crisis that will take time to resolve and therefore finding ways to address ECD and maternal mental health of the thousands of women and children is critical.

2. Integrated Innovation
Feedback: Combining ECD and maternal mental health supports/services to refugees is not necessarily a new idea but is still critical. This program aims to use an existing service (from Pakistan) and contextualize it to the needs/situation of refugee camps in Bangladesh.

3. Project Execution Plan
Feedback: The project execution plan is fairly well laid out. Additional information on how refinements to adapted curriculum might occur and how the community coordinators will be supported, provided refresher training beyond the 2 initial days would be worth considering.

4. Leadership Capability to Champion Change
Feedback: The teams seem experienced and capable to implement the program and the Bangladeshi NGO also appears to have good networks on the ground in the country - particularly in the health sector. The group of NGOs, government contacts, and funding organizations with whom they plan to share progress and impact should be useful in terms of local inputs and identifying pathways for scaling should the pilot show promise.

5. Value for Effort
Feedback: Budget seems reasonable - I have some concern that the support for the community workers implementing the services may be insufficient and that the 2 day training period may require some level of refresher training.

Reviewer 2

1. Impact
This addresses a very important issue in a particularly vulnerable community. If the intervention is effective, it can really help many women and children negatively affected by the trauma of systematic persecution, fleeing from their homes and losing loved ones due to violence.

2. Integrated Innovation
Much of the intervention is still yet to be designed so it is difficult to assess integrated innovation. Are there existing resources for refugees that can be adapted to this context? Will be interesting to see if the materials developed for a Pakistan context can be adapted successfully in a different country and in a very different context.

3. Project Execution Plan
Not sure if a two day training is sufficient. Managing 60 dyads is a lot for one person!
Scaling and sustainability is through govt scale up (Ministry of Health and Public Welfare, and Disaster Mgt and Relief) but they are not engaged in the program as much as WHO and UNICEF (who are their technical partners). Understanding how much buy-in there is from government agencies would be helpful.

4. Leadership Capability to Champion Change
Diverse and experienced leadership team with relevant experience in ECD and maternal health although none have experience in delivering these interventions in refugee settings...which could be a big challenge.

5. Value for Effort Reasonable.

Reviewer 3

This has significant potential.

Does need help with outcomes and method - some problems with method and outcomes but this could be improved

Took quite a long time before I could establish what the target age group here was
I think the team could be more familiar with Care for Child Development - not entirely clear that they know what care for child development is.

Not clear how many community health workers needed (l2 or ll) as there are ll intervention clusters. Not clear why it would take as long as 7 months to adapt materials? CCD has been adapted many times before in many contexts - there are a lot of data available on this process

Applicants need to engage with the issue of ethics - what are the ethics of not offering anything to the control arm.

Not clear that the primary outcome that they list is the best primary outcome. It is unlikely (I think) that a short intervention using CCD will improve motor skills in such a short time.

Scientific Officer
This proposal put forward by a Bangladesh based NGO (ARK) and a Pakistan based NGO (ASD, which is also a previous/current recipient of a GCC awards) aims to adapt and conceptualize an existing ECD and maternal mental health care and support model (from Pakistan) for Rohingya refugee women and children dyads who are based in selected camps in Bangladesh.

Strengths:
The project is worthwhile and highly ambitious.
The study design is clearly described and the costing exercise is well elaborated upon.
There is excellent potential for scaling up such an intervention both locally and in other settings. Moreover, the proposed intervention is based on a current intervention which will be modified to be more applicable to this population.

Weaknesses:
There is some concern expressed with respect to the background and experience and research skills of the applicants as well as their current connections/engagement with local stakeholders and community to initiate and be successful in implementing this project. However, this is acknowledged by the applicants and they propose a stakeholder analysis to help with project initiation. The measurement tools would require greater attention to ensure they are able to measure their desired outcomes. Perhaps securing more expertise in this area would strengthen the team.
More description of the content of the intervention over the two year period would be helpful. The two-day training period is perceived as likely insufficient and it is poorly described in the proposal as well they are encouraged to consider providing follow-up training and supervision as necessary.

Budget:

No issues were raised with respect to budget.
